# 2-(2,4-Dichloro­phen­yl)-9-phenyl-2,3-di­hydro­thieno[3,2-*b*]quinoline

**DOI:** 10.1107/S1600536809025380

**Published:** 2009-07-04

**Authors:** K. Balamurugan, D. Narmadha, J. Suresh, S. Perumal, P. L. Nilantha Lakshman

**Affiliations:** a School of Chemistry, Madurai Kamaraj University, Madurai 625 021, India; b Department of Physics, The Madura College, Madurai 625 011, India; c Department of Food Science and Technology, Faculty of Agriculture, University of Ruhuna, Mapalana, Kamburupitiya 81100, Sri Lanka

## Abstract

In the title compound, C_23_H_15_Cl_2_NS, the quinoline system is almost planar [r.m.s. deviation = 0.013 (2) Å]. The phenyl group is disordered over two positions with site occupancies of 0.55 and 0.45, and is oriented in a nearly perpendicular configuration to the quinoline ring [the dihedral angles between the quinoline ring and the major and minor disordered components of the phenyl ring are 81.8 (2) and 71.6 (2)°, respectively]. The dihydro­thiene ring adopts an envelope conformation. The dihedral angle between the chloro­phenyl ring and the quinoline system is 79.32 (1)°. In the crystal weak C—H⋯π inter­actions occur.

## Related literature

For the biological activity of quinoline derivatives, see: Kalluraya & Sreenivasa (1998 ▶); Maguire *et al.* (1994 ▶); Doube *et al.* (1998 ▶). For ring puckering analysis, see: Cremer & Pople (1975 ▶).
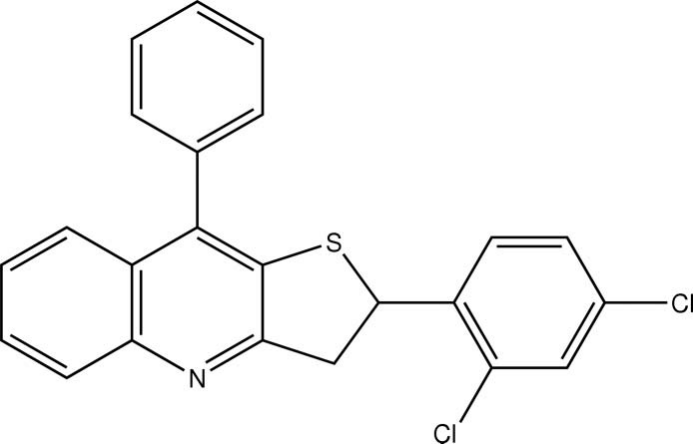


## Experimental

### 

#### Crystal data

C_23_H_15_Cl_2_NS
*M* *_r_* = 408.32
Monoclinic, 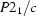

*a* = 11.8860 (5) Å
*b* = 11.5040 (5) Å
*c* = 14.0270 (6) Å
β = 94.297 (9)°
*V* = 1912.61 (14) Å^3^
*Z* = 4
Mo *K*α radiation
μ = 0.46 mm^−1^
*T* = 293 K
0.19 × 0.16 × 0.11 mm

#### Data collection

Nonius MACH-3 diffractometer
Absorption correction: ψ scan (North *et al.*, 1968 ▶) *T*_min_ = 0.917, *T*_max_ = 0.951
3917 measured reflections
3363 independent reflections
2577 reflections with *I* > 2σ(*I*)
*R*_int_ = 0.014
2 standard reflections frequency: 60 min intensity decay: none

#### Refinement

*R*[*F* ^2^ > 2σ(*F* ^2^)] = 0.032
*wR*(*F* ^2^) = 0.091
*S* = 1.02
3363 reflections
284 parameters
18 restraints
H atoms treated by a mixture of independent and constrained refinement
Δρ_max_ = 0.20 e Å^−3^
Δρ_min_ = −0.22 e Å^−3^

### 

Data collection: *CAD-4 EXPRESS* (Enraf–Nonius, 1994 ▶); cell refinement: *CAD-4 EXPRESS*; data reduction: *XCAD4* (Harms & Wocadlo, 1996 ▶); program(s) used to solve structure: *SHELXS97* (Sheldrick, 2008 ▶); program(s) used to refine structure: *SHELXL97* (Sheldrick, 2008 ▶); molecular graphics: *PLATON* (Spek, 2009 ▶); software used to prepare material for publication: *SHELXL97*.

## Supplementary Material

Crystal structure: contains datablocks global, I. DOI: 10.1107/S1600536809025380/at2782sup1.cif

Structure factors: contains datablocks I. DOI: 10.1107/S1600536809025380/at2782Isup2.hkl

Additional supplementary materials:  crystallographic information; 3D view; checkCIF report

## Figures and Tables

**Table 1:** Hydrogen-bond geometry (Å, °)

*D*—H⋯*A*	*D*—H	H⋯*A*	*D*⋯*A*	*D*—H⋯*A*
C8—H8⋯*Cg*2^i^	0.93	2.92	3.818 (2)	162
C21—H21⋯*Cg*3^ii^	0.93	2.71	3.636 (2)	172

Symmetry codes: (i) 
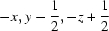
; (ii) 
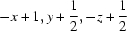
. *Cg2* and *Cg3* are the centroids of the N1/C2–C6 and C2/C3/C7–C10 rings, respectively.

**Fig. 1.**
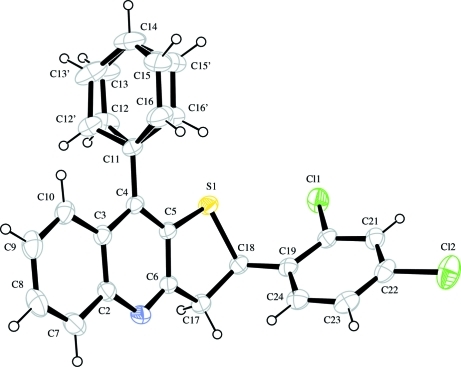
The molecular structure of the title compound, showing 30% probability displacement ellipsoids and the atom-numbering scheme. The two disorder components of the phenyl ring of the molecule are shown, the minor component is labeled with the suffix '. the disorder parts

## supplementary crystallographic information

### Comment

Quinoline exists as backbone in many natural products and pharmacologically important compounds. Their widerange of biological activities include antimalarial, antiasthmatic, antiinflamatory, antibacterial, antihypertensive and tyrosine kinase PDGF-RTK inhibiting agents (Kalluraya & Sreenivasa, 1998; Doube *et al.*, 1998; Maguire *et al.*, 1994). We report herein the synthesis and crystal structure of the title compound (I).

In the molecule of (I), (Fig. 1), the quinoline ring is planar and is oriented to the disordered phenyl ring in nearly perpendicular configuration. The dihendral angle between the major and minor components of the disordered phenyl rings is 26.6 (4)°. The dihydrothieno ring adopts envelope conformation with C18 being the flap atom. The puckering parameters are q_2_ = 0.333 (2) Å and φ_2_ = 319.7 (3)° (Cremer & Pople, 1975). The dihedral angle between the chlorophenyl ring and the quinoline ring is 79.32 (1)°.

In the crystal structure, there is no classical hydrogen bonds. The crystal packing is stabilized by two weak C—H···π interactions (Table 1; *Cg2* and *Cg3* refer to ring centroids of N1/C2–C6 and C2/C3/C7–C10, respectively).

### Experimental

A mixture of 5-(2,4-dichlorophenyl)dihydrothiophen-3(2*H*)-one, (1 mmol), 2-aminobenzophenone (1 mmol) and trifluroaceticacid (1.5 mmol) was taken in a 10 ml quartz vial and placed in the Biotage microwave oven. The vial was sealed and subjected to microwave irradiation. The irradiation was programmed at (273 K, 25 W, 0 bar, Absorption level: very high) for 30 min. (After a period of 1–2 min, the temperature reached a plateau, 273 K, and remained constant). After N_2_ gas jet cooling to room temperature (3 min), the reaction mixture was neutralized with NaHCO_3_ and extracted in CH_2_Cl_2_ (2 *X* 5 ml), dried over MgSO_4_ and concentrated *in vacuo* to give the crude product which was further purified either by a short column chromatography (silica gel, EtOAc-petroleumether, 2:8) to afford the corresponding pure quinoline derivative [melting point: 437–438 K, yield: 75%].

### Refinement

The disorder in the phenyl ring is identified as 'rotation disorder'. The phenyl ring is disordered over two orientations and it was resolved completely and their major and minor componenets have the site occupancies of 0.55 and 0.45.The bond distances in the ring is constrained using *DFIX* command. The bond distances and angles of the disordered ring are in agreement with normal phenyl rings. All H atoms of the disordered phenyl group were located in a difference Fourier map. The remaining H atoms were placed in calculated positions and allowed to ride on their carrier atoms with C—H = 0.93–0.98Å and *U*_iso_ = 1.2*U*_eq_(C) for CH,CH_2_ groups.

### Crystal data

**Table:**

C_23_H_15_Cl_2_NS	*F*(000) = 840
*M**_r_* = 408.32	*D*_x_ = 1.418 Mg m^−^^3^
Monoclinic, *P*2_1_/*c*	Mo *K*α radiation, λ = 0.71069 Å
Hall symbol: -P 2ybc	Cell parameters from 25 reflections
*a* = 11.8860 (5) Å	θ = 2–25°
*b* = 11.5040 (5) Å	µ = 0.46 mm^−^^1^
*c* = 14.0270 (6) Å	*T* = 293 K
β = 94.297 (9)°	Block, colourless
*V* = 1912.61 (14) Å^3^	0.19 × 0.16 × 0.11 mm
*Z* = 4	

### Data collection

**Table:**

Nonius MACH-3 diffractometer	2577 reflections with *I* > 2σ(*I*)
Radiation source: fine-focus sealed tube	*R*_int_ = 0.014
graphite	θ_max_ = 25.0°, θ_min_ = 2.3°
ω–2θ scans	*h* = 0→14
Absorption correction: ψ scan (North *et al.*, 1968)	*k* = −1→13
*T*_min_ = 0.917, *T*_max_ = 0.951	*l* = −16→16
3917 measured reflections	2 standard reflections every 60 min
3363 independent reflections	intensity decay: none

### Refinement

**Table:**

Refinement on *F*^2^	Secondary atom site location: difference Fourier map
Least-squares matrix: full	Hydrogen site location: inferred from neighbouring sites
*R*[*F*^2^ > 2σ(*F*^2^)] = 0.032	H atoms treated by a mixture of independent and constrained refinement
*wR*(*F*^2^) = 0.091	*w* = 1/[σ^2^(*F*_o_^2^) + (0.0458*P*)^2^ + 0.5943*P*] where *P* = (*F*_o_^2^ + 2*F*_c_^2^)/3
*S* = 1.02	(Δ/σ)_max_ < 0.001
3363 reflections	Δρ_max_ = 0.20 e Å^−^^3^
284 parameters	Δρ_min_ = −0.22 e Å^−^^3^
18 restraints	Extinction correction: *SHELXL97* (Sheldrick, 2008), Fc^*^=kFc[1+0.001xFc^2^λ^3^/sin(2θ)]^-1/4^
Primary atom site location: structure-invariant direct methods	Extinction coefficient: 0.0047 (10)

### Special details

**Table:**

Geometry. All e.s.d.'s (except the e.s.d. in the dihedral angle between two l.s. planes) are estimated using the full covariance matrix. The cell e.s.d.'s are taken into account individually in the estimation of e.s.d.'s in distances, angles and torsion angles; correlations between e.s.d.'s in cell parameters are only used when they are defined by crystal symmetry. An approximate (isotropic) treatment of cell e.s.d.'s is used for estimating e.s.d.'s involving l.s. planes.
Refinement. Refinement of *F*^2^ against ALL reflections. The weighted *R*-factor *wR* and goodness of fit *S* are based on *F*^2^, conventional *R*-factors *R* are based on *F*, with *F* set to zero for negative *F*^2^. The threshold expression of *F*^2^ > σ(*F*^2^) is used only for calculating *R*-factors(gt) *etc*. and is not relevant to the choice of reflections for refinement. *R*-factors based on *F*^2^ are statistically about twice as large as those based on *F*, and *R*- factors based on ALL data will be even larger.

### Fractional atomic coordinates and isotropic or equivalent isotropic displacement parameters (Å^2^)

**Table:**

	*x*	*y*	*z*	*U*_iso_*/*U*_eq_	Occ. (<1)
C2	0.11979 (16)	0.26004 (19)	0.25210 (14)	0.0464 (5)	
C3	0.14508 (15)	0.30797 (18)	0.34454 (13)	0.0428 (4)	
C4	0.21084 (15)	0.41146 (17)	0.35382 (12)	0.0405 (4)	
C5	0.24702 (14)	0.45739 (17)	0.27087 (12)	0.0395 (4)	
C6	0.21560 (15)	0.40461 (18)	0.18138 (12)	0.0428 (4)	
C7	0.05703 (19)	0.1566 (2)	0.24282 (17)	0.0629 (6)	
H7	0.0407	0.1247	0.1825	0.075*	
C8	0.01978 (19)	0.1025 (2)	0.3209 (2)	0.0701 (7)	
H8	−0.0219	0.0343	0.3134	0.084*	
C9	0.04406 (18)	0.1493 (2)	0.41213 (18)	0.0632 (6)	
H9	0.0185	0.1117	0.4651	0.076*	
C10	0.10473 (17)	0.2492 (2)	0.42434 (15)	0.0521 (5)	
H10	0.1199	0.2793	0.4855	0.063*	
C17	0.25242 (17)	0.4742 (2)	0.09826 (14)	0.0523 (5)	
H17A	0.1912	0.5235	0.0727	0.063*	
H17B	0.2735	0.4225	0.0479	0.063*	
C18	0.35345 (17)	0.54856 (19)	0.13425 (13)	0.0478 (5)	
H18	0.3512	0.6213	0.0977	0.057*	
C19	0.46829 (16)	0.49449 (17)	0.12880 (12)	0.0446 (5)	
C20	0.56613 (17)	0.55995 (18)	0.14608 (14)	0.0472 (5)	
C21	0.67332 (18)	0.5143 (2)	0.14224 (14)	0.0528 (5)	
H21	0.7369	0.5605	0.1549	0.063*	
C22	0.68341 (19)	0.3986 (2)	0.11907 (14)	0.0553 (5)	
C23	0.5893 (2)	0.3301 (2)	0.10080 (15)	0.0588 (6)	
H23	0.5971	0.2523	0.0847	0.071*	
C24	0.48327 (19)	0.37731 (19)	0.10646 (14)	0.0524 (5)	
H24	0.4202	0.3300	0.0951	0.063*	
N1	0.15524 (13)	0.31025 (16)	0.17022 (11)	0.0487 (4)	
Cl1	0.55600 (5)	0.70657 (5)	0.17491 (5)	0.0697 (2)	
Cl2	0.81734 (6)	0.33969 (7)	0.11513 (6)	0.0859 (3)	
S1	0.32820 (5)	0.58301 (5)	0.26012 (4)	0.05025 (17)	
C11	0.23924 (16)	0.46862 (18)	0.44796 (13)	0.0453 (5)	
C14	0.2954 (3)	0.5830 (3)	0.61987 (18)	0.0854 (9)	
H14	0.312 (2)	0.624 (3)	0.678 (2)	0.102*	
C12	0.1794 (7)	0.5609 (6)	0.4804 (6)	0.067 (2)	0.55
H12	0.1176	0.5866	0.4415	0.080*	0.55
C13	0.2032 (7)	0.6189 (8)	0.5661 (6)	0.085 (3)	0.55
H13	0.1581	0.6792	0.5854	0.102*	0.55
C15	0.3566 (7)	0.4863 (7)	0.5933 (7)	0.094 (3)	0.55
H15	0.4163	0.4594	0.6340	0.112*	0.55
C16	0.3298 (6)	0.4293 (8)	0.5068 (6)	0.076 (3)	0.55
H16	0.3722	0.3660	0.4890	0.091*	0.55
C12'	0.1533 (9)	0.5268 (6)	0.4904 (7)	0.055 (2)	0.45
H12'	0.0794	0.5282	0.4637	0.066*	0.45
C13'	0.1868 (8)	0.5831 (8)	0.5765 (7)	0.075 (3)	0.45
H13'	0.1319	0.6236	0.6068	0.090*	0.45
C15'	0.3783 (9)	0.5283 (9)	0.5745 (8)	0.083 (3)	0.45
H15'	0.4527	0.5289	0.6004	0.099*	0.45
C16'	0.3486 (7)	0.4717 (9)	0.4887 (7)	0.064 (3)	0.45
H16'	0.4048	0.4344	0.4574	0.077*	0.45

### Atomic displacement parameters (Å^2^)

**Table:**

	*U*^11^	*U*^22^	*U*^33^	*U*^12^	*U*^13^	*U*^23^
C2	0.0389 (10)	0.0567 (12)	0.0433 (10)	−0.0048 (9)	−0.0001 (8)	0.0028 (9)
C3	0.0359 (9)	0.0532 (12)	0.0393 (10)	0.0020 (9)	0.0028 (7)	0.0059 (9)
C4	0.0377 (9)	0.0506 (11)	0.0334 (9)	0.0059 (9)	0.0035 (7)	0.0029 (8)
C5	0.0373 (9)	0.0471 (10)	0.0340 (9)	0.0011 (8)	0.0030 (7)	0.0011 (8)
C6	0.0387 (9)	0.0563 (12)	0.0332 (9)	−0.0003 (9)	0.0017 (7)	0.0014 (9)
C7	0.0544 (13)	0.0730 (16)	0.0599 (13)	−0.0185 (12)	−0.0042 (10)	−0.0018 (12)
C8	0.0527 (13)	0.0715 (16)	0.0858 (18)	−0.0230 (12)	0.0033 (12)	0.0093 (14)
C9	0.0502 (12)	0.0736 (16)	0.0670 (15)	−0.0064 (12)	0.0114 (11)	0.0221 (12)
C10	0.0467 (11)	0.0646 (13)	0.0459 (11)	0.0017 (10)	0.0092 (9)	0.0101 (10)
C17	0.0532 (11)	0.0699 (14)	0.0339 (9)	−0.0036 (11)	0.0038 (8)	0.0064 (10)
C18	0.0537 (11)	0.0552 (12)	0.0353 (9)	−0.0033 (10)	0.0087 (8)	0.0079 (9)
C19	0.0540 (11)	0.0492 (11)	0.0314 (9)	−0.0030 (9)	0.0089 (8)	0.0074 (8)
C20	0.0554 (12)	0.0465 (11)	0.0410 (10)	−0.0023 (9)	0.0124 (9)	0.0054 (8)
C21	0.0524 (12)	0.0611 (14)	0.0461 (11)	−0.0019 (10)	0.0118 (9)	0.0094 (10)
C22	0.0631 (13)	0.0604 (14)	0.0443 (11)	0.0125 (11)	0.0168 (10)	0.0137 (10)
C23	0.0842 (17)	0.0479 (12)	0.0456 (11)	0.0063 (12)	0.0138 (11)	0.0070 (10)
C24	0.0649 (13)	0.0514 (12)	0.0416 (10)	−0.0075 (10)	0.0077 (9)	0.0049 (9)
N1	0.0466 (9)	0.0625 (11)	0.0364 (8)	−0.0067 (8)	−0.0004 (7)	−0.0022 (8)
Cl1	0.0660 (4)	0.0495 (3)	0.0953 (5)	−0.0083 (3)	0.0177 (3)	−0.0065 (3)
Cl2	0.0773 (4)	0.0853 (5)	0.0990 (5)	0.0299 (4)	0.0315 (4)	0.0238 (4)
S1	0.0578 (3)	0.0512 (3)	0.0430 (3)	−0.0080 (2)	0.0117 (2)	−0.0035 (2)
C11	0.0528 (11)	0.0530 (12)	0.0303 (9)	−0.0003 (10)	0.0046 (8)	0.0038 (9)
C14	0.132 (3)	0.085 (2)	0.0384 (13)	−0.019 (2)	0.0008 (16)	−0.0091 (14)
C12	0.086 (5)	0.047 (4)	0.064 (4)	0.013 (3)	−0.018 (3)	−0.007 (3)
C13	0.148 (7)	0.041 (5)	0.062 (5)	0.015 (4)	−0.022 (4)	−0.009 (3)
C15	0.076 (5)	0.158 (9)	0.043 (4)	0.005 (5)	−0.016 (3)	−0.007 (5)
C16	0.070 (4)	0.112 (7)	0.044 (3)	0.031 (4)	−0.003 (3)	−0.009 (4)
C12'	0.077 (5)	0.048 (5)	0.042 (3)	0.002 (4)	0.012 (3)	0.000 (3)
C13'	0.144 (8)	0.033 (5)	0.050 (4)	0.024 (4)	0.028 (5)	0.001 (3)
C15'	0.083 (5)	0.113 (7)	0.051 (6)	−0.027 (5)	−0.005 (4)	−0.012 (5)
C16'	0.060 (4)	0.088 (7)	0.044 (5)	−0.007 (4)	−0.001 (3)	−0.010 (4)

### Geometric parameters (Å, °)

**Table:**

C2—N1	1.379 (2)	C21—C22	1.378 (3)
C2—C7	1.405 (3)	C21—H21	0.9300
C2—C3	1.420 (3)	C22—C23	1.376 (3)
C3—C10	1.421 (3)	C22—Cl2	1.735 (2)
C3—C4	1.425 (3)	C23—C24	1.380 (3)
C4—C5	1.376 (2)	C23—H23	0.9300
C4—C11	1.491 (3)	C24—H24	0.9300
C5—C6	1.419 (3)	C11—C12	1.374 (7)
C5—S1	1.7505 (19)	C11—C16'	1.381 (8)
C6—N1	1.304 (2)	C11—C16	1.383 (7)
C6—C17	1.506 (3)	C11—C12'	1.391 (8)
C7—C8	1.362 (3)	C14—C13	1.348 (7)
C7—H7	0.9300	C14—C15'	1.366 (8)
C8—C9	1.399 (4)	C14—C13'	1.385 (8)
C8—H8	0.9300	C14—C15	1.395 (7)
C9—C10	1.361 (3)	C14—H14	0.95 (3)
C9—H9	0.9300	C12—C13	1.385 (7)
C10—H10	0.9300	C12—H12	0.9300
C17—C18	1.529 (3)	C13—H13	0.9300
C17—H17A	0.9700	C15—C16	1.395 (7)
C17—H17B	0.9700	C15—H15	0.9300
C18—C19	1.507 (3)	C16—H16	0.9300
C18—S1	1.8557 (19)	C12'—C13'	1.401 (8)
C18—H18	0.9800	C12'—H12'	0.9300
C19—C20	1.391 (3)	C13'—H13'	0.9300
C19—C24	1.398 (3)	C15'—C16'	1.391 (8)
C20—C21	1.383 (3)	C15'—H15'	0.9300
C20—Cl1	1.741 (2)	C16'—H16'	0.9300
			
N1—C2—C7	118.08 (18)	C22—C23—H23	120.1
N1—C2—C3	122.72 (18)	C24—C23—H23	120.1
C7—C2—C3	119.20 (18)	C23—C24—C19	121.6 (2)
C2—C3—C10	118.26 (19)	C23—C24—H24	119.2
C2—C3—C4	119.01 (16)	C19—C24—H24	119.2
C10—C3—C4	122.72 (18)	C6—N1—C2	116.63 (16)
C5—C4—C3	116.60 (16)	C5—S1—C18	92.02 (9)
C5—C4—C11	121.01 (18)	C12—C11—C16'	109.8 (7)
C3—C4—C11	122.39 (16)	C12—C11—C16	117.0 (6)
C4—C5—C6	120.37 (18)	C16'—C11—C16	25.1 (5)
C4—C5—S1	126.81 (15)	C12—C11—C12'	21.9 (5)
C6—C5—S1	112.78 (13)	C16'—C11—C12'	120.6 (7)
N1—C6—C5	124.62 (17)	C16—C11—C12'	117.7 (6)
N1—C6—C17	122.59 (17)	C12—C11—C4	123.0 (4)
C5—C6—C17	112.65 (17)	C16'—C11—C4	121.4 (5)
C8—C7—C2	121.0 (2)	C16—C11—C4	119.9 (4)
C8—C7—H7	119.5	C12'—C11—C4	117.8 (5)
C2—C7—H7	119.5	C13—C14—C15'	117.7 (7)
C7—C8—C9	120.2 (2)	C13—C14—C13'	20.3 (6)
C7—C8—H8	119.9	C15'—C14—C13'	118.5 (7)
C9—C8—H8	119.9	C13—C14—C15	120.9 (6)
C10—C9—C8	120.7 (2)	C15'—C14—C15	25.8 (6)
C10—C9—H9	119.6	C13'—C14—C15	111.8 (6)
C8—C9—H9	119.6	C13—C14—H14	116.3 (19)
C9—C10—C3	120.6 (2)	C15'—C14—H14	120.9 (19)
C9—C10—H10	119.7	C13'—C14—H14	120.5 (19)
C3—C10—H10	119.7	C15—C14—H14	122.7 (19)
C6—C17—C18	107.96 (16)	C11—C12—C13	125.6 (8)
C6—C17—H17A	110.1	C11—C12—H12	117.2
C18—C17—H17A	110.1	C13—C12—H12	117.2
C6—C17—H17B	110.1	C14—C13—C12	116.3 (8)
C18—C17—H17B	110.1	C14—C13—H13	121.9
H17A—C17—H17B	108.4	C12—C13—H13	121.9
C19—C18—C17	116.36 (18)	C14—C15—C16	121.2 (8)
C19—C18—S1	110.37 (13)	C14—C15—H15	119.4
C17—C18—S1	104.75 (12)	C16—C15—H15	119.4
C19—C18—H18	108.4	C11—C16—C15	118.8 (8)
C17—C18—H18	108.4	C11—C16—H16	120.6
S1—C18—H18	108.4	C15—C16—H16	120.6
C20—C19—C24	116.24 (19)	C11—C12'—C13'	114.9 (9)
C20—C19—C18	121.07 (18)	C11—C12'—H12'	122.5
C24—C19—C18	122.69 (18)	C13'—C12'—H12'	122.5
C21—C20—C19	123.2 (2)	C14—C13'—C12'	124.9 (9)
C21—C20—Cl1	117.18 (16)	C14—C13'—H13'	117.5
C19—C20—Cl1	119.57 (16)	C12'—C13'—H13'	117.5
C22—C21—C20	118.2 (2)	C14—C15'—C16'	118.3 (11)
C22—C21—H21	120.9	C14—C15'—H15'	120.9
C20—C21—H21	120.9	C16'—C15'—H15'	120.9
C23—C22—C21	120.9 (2)	C11—C16'—C15'	122.7 (11)
C23—C22—Cl2	120.38 (18)	C11—C16'—H16'	118.7
C21—C22—Cl2	118.75 (19)	C15'—C16'—H16'	118.7
C22—C23—C24	119.8 (2)		
			
N1—C2—C3—C10	−179.85 (18)	C7—C2—N1—C6	−178.41 (19)
C7—C2—C3—C10	−0.5 (3)	C3—C2—N1—C6	1.0 (3)
N1—C2—C3—C4	−0.7 (3)	C4—C5—S1—C18	168.69 (17)
C7—C2—C3—C4	178.65 (19)	C6—C5—S1—C18	−13.48 (15)
C2—C3—C4—C5	−0.9 (3)	C19—C18—S1—C5	−99.75 (15)
C10—C3—C4—C5	178.20 (17)	C17—C18—S1—C5	26.24 (15)
C2—C3—C4—C11	178.78 (17)	C5—C4—C11—C12	82.4 (4)
C10—C3—C4—C11	−2.1 (3)	C3—C4—C11—C12	−97.3 (4)
C3—C4—C5—C6	2.2 (3)	C5—C4—C11—C16'	−68.1 (5)
C11—C4—C5—C6	−177.50 (17)	C3—C4—C11—C16'	112.3 (5)
C3—C4—C5—S1	179.84 (14)	C5—C4—C11—C16	−97.3 (5)
C11—C4—C5—S1	0.2 (3)	C3—C4—C11—C16	83.0 (5)
C4—C5—C6—N1	−2.1 (3)	C5—C4—C11—C12'	107.1 (4)
S1—C5—C6—N1	179.96 (16)	C3—C4—C11—C12'	−72.5 (4)
C4—C5—C6—C17	173.78 (17)	C16'—C11—C12—C13	−25.1 (9)
S1—C5—C6—C17	−4.2 (2)	C16—C11—C12—C13	1.1 (9)
N1—C2—C7—C8	179.8 (2)	C12'—C11—C12—C13	99 (2)
C3—C2—C7—C8	0.4 (3)	C4—C11—C12—C13	−178.6 (6)
C2—C7—C8—C9	−0.2 (4)	C15'—C14—C13—C12	24.1 (10)
C7—C8—C9—C10	0.1 (4)	C13'—C14—C13—C12	−74 (2)
C8—C9—C10—C3	−0.2 (3)	C15—C14—C13—C12	−5.3 (10)
C2—C3—C10—C9	0.4 (3)	C11—C12—C13—C14	2.3 (11)
C4—C3—C10—C9	−178.69 (19)	C13—C14—C15—C16	5.1 (12)
N1—C6—C17—C18	−159.49 (18)	C15'—C14—C15—C16	−85 (2)
C5—C6—C17—C18	24.6 (2)	C13'—C14—C15—C16	25.4 (11)
C6—C17—C18—C19	90.0 (2)	C12—C11—C16—C15	−1.5 (10)
C6—C17—C18—S1	−32.2 (2)	C16'—C11—C16—C15	77 (2)
C17—C18—C19—C20	170.37 (17)	C12'—C11—C16—C15	−26.2 (10)
S1—C18—C19—C20	−70.5 (2)	C4—C11—C16—C15	178.2 (6)
C17—C18—C19—C24	−9.6 (3)	C14—C15—C16—C11	−1.4 (12)
S1—C18—C19—C24	109.51 (18)	C12—C11—C12'—C13'	−67 (2)
C24—C19—C20—C21	−0.2 (3)	C16'—C11—C12'—C13'	−1.9 (9)
C18—C19—C20—C21	179.80 (17)	C16—C11—C12'—C13'	26.7 (8)
C24—C19—C20—Cl1	−179.75 (14)	C4—C11—C12'—C13'	−177.2 (5)
C18—C19—C20—Cl1	0.2 (2)	C13—C14—C13'—C12'	96 (3)
C19—C20—C21—C22	0.8 (3)	C15'—C14—C13'—C12'	2.8 (12)
Cl1—C20—C21—C22	−179.67 (15)	C15—C14—C13'—C12'	−24.8 (10)
C20—C21—C22—C23	−0.4 (3)	C11—C12'—C13'—C14	−0.6 (11)
C20—C21—C22—Cl2	−179.36 (15)	C13—C14—C15'—C16'	−25.4 (12)
C21—C22—C23—C24	−0.6 (3)	C13'—C14—C15'—C16'	−2.3 (12)
Cl2—C22—C23—C24	178.41 (15)	C15—C14—C15'—C16'	78.8 (19)
C22—C23—C24—C19	1.2 (3)	C12—C11—C16'—C15'	23.5 (10)
C20—C19—C24—C23	−0.8 (3)	C16—C11—C16'—C15'	−88 (2)
C18—C19—C24—C23	179.23 (18)	C12'—C11—C16'—C15'	2.3 (11)
C5—C6—N1—C2	0.4 (3)	C4—C11—C16'—C15'	177.4 (7)
C17—C6—N1—C2	−175.03 (18)	C14—C15'—C16'—C11	−0.1 (14)

### Hydrogen-bond geometry (Å, °)

**Table:**

*D*—H···*A*	*D*—H	H···*A*	*D*···*A*	*D*—H···*A*
C8—H8···Cg2^i^	0.93	2.92	3.818 (2)	162
C21—H21···Cg3^ii^	0.93	2.71	3.636 (2)	172

Symmetry codes: (i) −*x*, *y*−1/2, −*z*+1/2; (ii) −*x*+1, *y*+1/2, −*z*+1/2.
